# Complete Genome Sequences of Two *Citrobacter rodentium* Bacteriophages, CR8 and CR44b

**DOI:** 10.1128/genomeA.00146-14

**Published:** 2014-05-29

**Authors:** Ana Luisa Toribio, Derek Pickard, Ana M. Cerdeño-Tárraga, Nicola K. Petty, Nicholas Thomson, George Salmond, Gordon Dougan

**Affiliations:** a The Wellcome Trust Sanger Institute, Wellcome Trust Genome Campus, Hinxton, Cambridgeshire, United Kingdom; b Department of Biochemistry, University of Cambridge, Cambridge, United Kingdom

## Abstract

The complete genomes of two virulent phages infecting *Citrobacter rodentium* are reported here for the first time. Both bacteriophages were isolated from local sewage treatment plant effluents. Genome analyses revealed a close relationship between both phages and allowed their classification as members of the *Autographivirinae* subfamily in the T7-like genus.

## GENOME ANNOUNCEMENT

We present here the first genome sequences of two virulent phages of *Citrobacter rodentium*, a natural murine enteropathogen that serves as an important model for enteropathogenic and enterohemorrhagic *Escherichia coli* infection, bowel inflammation, and immunity (1–3). The *C. rodentium* ICC168 genome has been sequenced, revealing a significant genetic relationship with *E. coli* (4, 5) and the identification of a number of prophages (6). We previously reported the characterization of a virulent generalized transducing phage for *C. rodentuim*, phage CR1 (7). However, the genome of this phage was recalcitrant to sequencing, and there is limited information about other virulent phages infecting *C. rodentium*.

Phages CR8 and CR44b were isolated from the effluent of a local sewage treatment plant in Cambridgeshire, United Kingdom. They are virulent phages for *C. rodentium* ICC168. While CR8 grows only at 25°C, CR44b presents similar efficiency of plating at both 37°C and 25°C. Phage DNA sequencing was performed at the Pathogen Sequencing Unit of the Wellcome Trust Sanger Institute from DNA extracted from CsCl-purified virion phage particles, using a shotgun approach and extensive primer walking. The sequences of CR8 and CR44b are 39,651 and 39,207 bp in length, respectively, including the terminal direct repeats (189 and 182 bp, respectively, while T7 phage presents 160 bp terminal repeats). Both genomes follow the well-conserved genomic organization of the T7-like phages. The *in silico* analysis revealed the existence of 58 predicted coding sequences (CDSs) for CR8 and 59 CDSs for CR44b, including one and two pseudogenes, respectively. The annotated pseudogene in CR8 (locus CR8_57) corresponds to a duplication of the gene 19.2; Gp 19.2 is a T7-like conserved phage protein potentially present in both phages (loci CR44b_57 and CR8_56). In the CR44b phage genome, the locus CR44b_01 describes an additional pseudogene that is also a conserved phage protein, though it was absent in this case from both the CR8 and T7 phages. Finally, the locus CR44b_12 contains a pseudogene of a T7 homologue (gene 1.7), which is not present in CR8.

In both phage genomes, 55% of the predicted CDSs were assigned a function, while >25% of the proteins were not functionally assigned but were found to be conserved in some other phage genomes. A unique feature shared by these phages is the presence of a putative tailspike (loci CR8_49 and CR44b_50), with no obvious sequence similarities to similar genes in databases, but these proteins present predicted folding with similarity to the adsorption protein p2 of phage PRD1, analyzed using Phyre (8). Another difference between the genomes is the absence in CR44b of the T7 gene 0.7 (protein kinase) homologue. The genomic sequences of these two phages will help shed light on the evolution of the T7-like phages and of their bacterium-targeting properties. These sequenced phages might also be used in investigations into phage-bacterium interactions.

### Nucleotide sequence accession numbers.

The genome sequences of CR44b and CR8 have been deposited in the ENA under the accession no. HG818823 and HG818824, respectively.
